# Inflammasome in Intestinal Inflammation and Cancer

**DOI:** 10.1155/2013/654963

**Published:** 2013-03-28

**Authors:** Tiago Nunes, Heitor S. de Souza

**Affiliations:** ^1^Biofunctionality Unit, ZIEL Research Center for Nutrition and Food Sciences, Technical University of Munich, 85354 Freising-Weihenstephan, Germany; ^2^Serviço de Gastroenterologia & Laboratório Multidisciplinar de Pesquisa, Hospital Universitario, Universidade Federal do Rio de Janeiro, 21941-913 Rio de Janeiro, RJ, Brazil

## Abstract

The activation of specific cytosolic pathogen recognition receptors, the nucleotide-binding-oligomerization-domain- (NOD-) like receptors (NLRs), leads to the assembly of the inflammasome, a multimeric complex platform that activates caspase-1. The caspase-1 pathway leads to the upregulation of important cytokines from the interleukin (IL)-1 family, IL-1**β**, and IL-18, with subsequent activation of the innate immune response. In this review, we discuss the molecular structure, the mechanisms behind the inflammasome activation, and its possible role in the pathogenesis of inflammatory bowel diseases and intestinal cancer. Here, we show that the available data points towards the importance of the inflammasome in the innate intestinal immune response, being the complex involved in the maintenance of intestinal homeostasis, correct intestinal barrier function and efficient elimination of invading pathogens.

## 1. Introduction

In the human gut, trillions of bacteria interact with the host's systemic immune system in a complex balance between immune activation and tolerance [1]. Pathogen recognition receptors (PRRs) have been shown to play an important role in the differentiation between commensal and pathogenic bacteria [2]. The detection of pathogen molecules collectively known as pathogen-associated molecular patterns (PAMPs) by PRRs activates the innate immune system, being involved in infection recognition and its consequent inflammatory response [2, 3]. The activation of PRRs can lead to enhanced production of proinflammatory cytokines with a wide range of systemic and local effects. Among them, interleukin (IL)-1*β* has been shown to be secreted in high amounts by colonic monocytes from patients with active inflammatory bowel disease (IBD), and intestinal levels are consistently correlated with disease activity suggesting an important role of this cytokine in intestinal inflammation [4]. In addition, IL-1 has also been implicated in the promotion of angiogenesis, tumor growth, and metastasis in experimental cancer models, being associated with more aggressive tumor biology [5, 6].

Generation of IL-1*β* requires the activity of caspase-1, but the mechanism involved in the activation of proinflammatory caspases remained to be established until 2002. In that year, the group of the late Jürg Tschopp reported the identification of the inflammasome, a multimeric molecular platform which triggers the activation of inflammatory caspases and processes pro-IL-1*β* [13]. Inflammasomes are cytosolic multiprotein complexes activated by specific PRRs which are involved in infection recognition and inflammation [13–17]. The structure of the inflammasome is assembled by intracellular nucleotide-binding-oligomerization-domain- (NOD-) like receptors (NLRs) to initiate innate immune responses against invading pathogens activating caspase-l [15]. The subsequent activation of caspase-l leads to enhanced expression of IL-1*β* and IL-18, recruitment and activation of immune cells, and triggering of pyroptosis, a caspase-1-dependent inflammatory form of cell death [14, 18–21].

The importance of the inflammasome and the cell death programming associated with microbial invasion is to restrict pathogen growth and to activate and recruit immune cells to mediate host defense. As the activation of the inflammasome and the caspase-1 pathway leads to enhanced production of cytokines known to be upregulated in IBD and cancer, the role of this pathway in intestinal inflammation and colonic neoplasia has been the subject of intense research in recent years.

## 2. The Inflammasome

**Figure 1:**
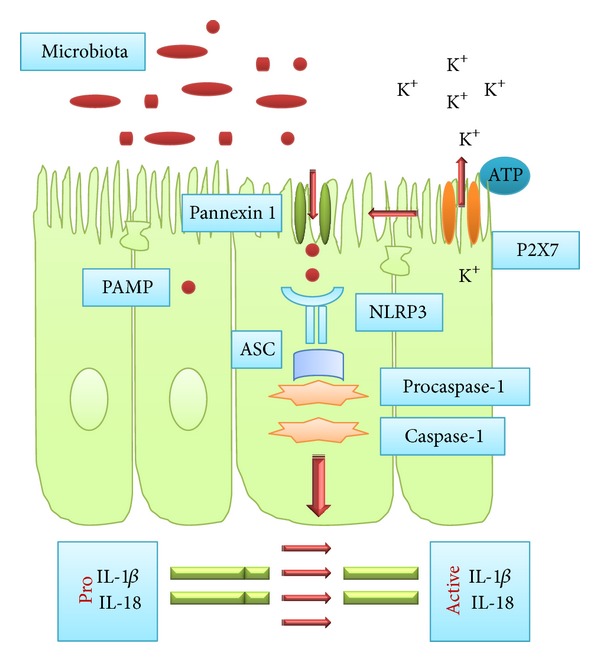
Inflammasome components, assembly, and activation. Microbial and nonmicrobial stimuli can induce the activation of the NLRP3 inflammasome. The NLRP3 inflammasome consists of a nucleotide-binding-oligomerization-domain- (NOD-) like receptor (NLR) that can be activated by certain bacterial toxins containing specific pathogen-associated molecular patterns (PAMPs), in the presence of extracellular adenosine triphosphate (ATP). Oligomerization of the NLR and ASC results in a macromolecular complex capable of cleaving procaspase-1 to its active form, which in turn cleaves the proforms of IL-1*β* and IL-18 to their biologically active forms.

Inflammasomes are composed of multiprotein cytosolic complexes that gather to activate caspase-1 [13]. These multimeric platforms are found in a wide range of cell types including macrophages, dendritic cells, adipocytes, keratinocytes, and epithelial cells [22–28]. These complexes are activated either by NLR proteins NLRP1, NLRP3, NLRC4, NLRP6, and NAIP5 or by the DNA-sensing complex of AIM2, a member of the interferon-inducible HIN-200 protein family. Activation of these receptors by certain PAMPs leads to their oligomerization and subsequent interaction with the adaptor protein ASC and the CARD domain of caspase-1. ASC, as well, presents a CARD domain that works together with the CARD domain of procaspase-1 [15]. Inflammasome-activated caspase-1 is then used for activation of the proinflammatory cytokines IL-1*β* and IL-18, both belonging to the IL-1 family. These inflammatory cytokines enhance antimicrobial functions of phagosomes and promote protection against intracellular pathogens [16] (Figure 1).

## 3. Inflammasome and Inflammatory Bowel Disease

### 3.1. The Association between the Inflammasome and Inflammatory Bowel Diseases

Crohn's disease (CD) and ulcerative colitis (UC) are chronic immune-mediated inflammatory diseases of the gastrointestinal tract that result from a dysregulated mucosal immune response to bacterial antigens in the gut lumen of a genetically susceptible host [29, 30]. In the gut-microbiota interplay related to IBD pathogenesis, several previous findings point towards the potential role of the inflammasome in the development of chronic intestinal inflammation. The first evidence refers to the upregulation of inflammatory cytokines IL-1*β* and IL-18 in active IBD, and the discovery of IL-18 gene polymorphisms associated with CD [31–33]. The second is the presence of a dysregulated IL-1*β* production linked to CD and the association between the NLRP3 inflammasome and three rare autoinflammatory chronic disorders treated with Canakinumab, a human monoclonal antibody targeted at IL-1*β* [22, 31, 34, 35]. The third, and perhaps the most important evidence, is the association between the NLRP3 gene and CD in candidate-gene approach studies. 

Genome-wide association studies (GWAS) have tried to dissect the inherited element of IBD, identifying more than 70 CD and 40 UC susceptibility loci [36, 37]. These studies, however, do not explain the majority of the heritability related to IBD [38]. One interesting genomic region not associated with IBD in GWAS, but pointed out in candidate-gene approach studies and gene expression analysis data, is the NLRP3 gene which encodes the NLRP3 or cryopyrin protein [39–41]. This protein is part of the NLRP3-inflammasome, and it has a pivotal role in the pathogenesis of other chronic inflammatory disorders as pseudogout, gout, and familial Mediterranean fever [42–44]. As a result of the genetic link between NLRP3 and CD, the NLRP3 inflammasome is the most studied caspase-1 inductor multimeric platform in the field of chronic intestinal inflammation. 

In two independent candidate-gene studies, the NLRP3 gene was associated with CD [39, 40], but there was no association in a posterior large study from the UK [45]. Interestingly, the first study conducted by Villani and coworkers also performed functional assays to evaluate the impact of these polymorphisms in NLRP3 expression and IL-1*β* production [39]. In this regard, NLRP3 SNPs were associated with lower levels of NLRP3 m-RNA expression in a loss-of-function fashion with homozygosis for the risk allele being associated with the lowest level of NLRP3 expression in peripheral blood cells and monocytes. In addition, they observed an association between lower IL-1*β* levels and the risk NLRP3 allele in cultured monocytes in the presence or absence of lipopolysaccharide. In both cases, homozygosis for the risk allele was associated with the lowest level of IL-1*β*. Even though significantly higher IL-1*β* levels were found in the ulcerated intestinal mucosa from human CD samples than in healthy controls, it was postulated that a dysregulated IL-1*β* production might play a role in CD pathogenesis for patients bearing these SNPs.

The second study identifying an association between polymorphisms in the NLRP3 and CD included 498 cases and 794 controls, reporting that variants of NLRP3 conferred susceptibility to CD in Swedish male individuals [40]. Even though an NLRP3 genetic susceptibility was found in this population, careful analysis of the results show that, differently from the study by Villani and coworkers, the NLRP3 SNP associated with CD was a gain-of-function polymorphism, possibly promoting the production of mature IL-1*β* with subsequent induction of caspase-1 activity. The authors postulate that patients with this specific NLRP3 polymorphism might present an increased susceptibility to CD as a result of an increased IL-1*β* production and not due to a dysregulation of the pathway. In addition, the risk for developing CD in this study was exclusively associated with male patients bearing variant alleles in both NLRP3 and CARD8 genes. A third study evaluating the association between NLRP3 polymorphisms and IBD added more controversy to the topic. Lewis and coworkers raised questions about the previously reported association between CD and the NLRP3 locus supported by negative results based on control allele frequency data from large GWA studies [45]. 

### 3.2. Inflammasome Activation in the Gut

Even though the role of the NLRP3 inflammasome in IBD is still a matter of debate, the mechanisms behind its function started to be recently unveiled. NLRP3 can be triggered by bacterial constituents, synthetic purine-like compounds, endogenous urate crystals, and exogenous adenosine triphosphate (ATP) [46–48]. Of note, it was postulated that the passage of bacterial molecules into the host cytosol leading to NLRP3 inflammasome activation can be mediated by pannexin-1 and P2X_7_ receptor [49]. Pannexin-1 constitutes a transmembrane hemichannel that associates with P2X_7_ receptor, a member of the ATP-activated P2X purinergic receptors family, permeable to monovalent cations and anions, and capable of inducing the opening of a larger pore permeable to hydrophilic macromolecules [50]. In particular, the P2X_7_ receptors act as danger sensors in immune cells and have been implicated in different biological functions, including apoptosis and the production and release of proinflammatory cytokines [51]. 

In this regard, investigators have demonstrated that the cytosolic recognition of bacterial molecules resulting in the NLRP3 inflammasome activation is mediated by pannexin-1 activation [52]. These results seem to indicate that NLRP3 would function downstream of pannexin-1/P2X_7_ receptor in response to bacterial components to regulate caspase-1 activation (Figure 1). Furthermore, the expression and site-specific modulation of P2X_7_ receptors was demonstrated on epithelial and immune cells of the gut, supporting the suggestion of purinergic signaling as an additional component of the innate immune circuits involved in the control of inflammation and cell fate in the gut and gut-associated lymphoid tissues [53]. In addition, in intestinal epithelial cells, the expression of P2X_7_ receptors was also found to be upregulated by interferon-gamma, a proinflammatory cytokine and a signature molecule of the Th-1 type of immune response [54]. Moreover, ATP was shown to induce apoptosis and autophagy in human epithelial cells, possibly via reactive oxygen species production, through activation of the P2X_7_ receptor [55]. Taken together, these findings appear to implicate P2X_7_ receptors associated with pannexin-1 and the consequent NLRP3 inflammasome activation in the pathogenesis of diseases based on the dysregulation of the immune response such as IBD.

### 3.3. Inflammasome and Intestinal Inflammation in Animal Models

As the main downstream impact of the activation of the inflammasome is the upregulation of IL-18 and IL-1*β*, the knockout of these two important inflammatory cytokines as well as of other upstream regulators is pivotal to fully understand the role of the inflammasome in intestinal inflammation. Therefore, genetically modified mice lacking IL-18, IL-18 receptor (IL-18R), IL-1 receptor (IL-1R), NLRP3, NLRP6, ASC, and caspase-1 were constructed. In general, the susceptibility of these animals to intestinal inflammation was tested using the dextran sulphate sodium (DSS) experimental colitis model.

In the context of DSS colitis models, the role of IL-18 and IL-1*β* is still a matter of debate. More recently, it has been shown that IL-18 and IL-18R knockout mice develop more severe inflammation compared to wild-type, which is not true for IL-1R knockout mice [56, 57]. In an infection mouse model with *C. rodentium*, however, IL-1R knockout was shown to present increase mortality with severe colitis characterized by intramural colonic bleeding and intestinal damage following infection [57]. Consistent with these findings, most recent studies present clear data that mice lacking NLRP3 are more susceptible to develop colitis [26, 58–60] and ASC and caspase-1-deficient mice present enhanced susceptibility to DSS-induced inflammation [61].

However, other studies have shown exactly the opposite—that transgenic or pharmacological blockage of IL-1*β* converting enzyme (ICE) or IL-18 ameliorate DSS colitis [62–65]. In keeping with these results, Bauer and coworkers reported decreased sensitivity to DSS in NLRP3 deficient mice [66]. In that study, IL-1*β* secretion was abrogated in macrophages lacking NLRP3, ASC, or caspase-1 confirming that DSS activates caspase-1 via the NLRP3 inflammasome. After administration of DSS, NLRP3 knockout mice developed less severe colitis than wild-type mice and produced lower levels of proinflammatory cytokines in colonic tissue. In addition, pharmacological inhibition of caspase-1 with pralnacasan achieved a level of mucosal protection equivalent to NLRP3 deficiency. More recently, this protective role of NLRP3 against DSS colitis was also demonstrated by yet another independent group [67]. In any case, regardless of the still debated role of the NLRP3 inflammasome in DSS colitis, it has been shown that NLRP3, ASC, and caspase-1 deficient mice do not develop colitis without DSS treatment, implying that isolated inflammasome impairment does not result in spontaneous intestinal inflammation [61].

Another inflammasome, NLRP6 [68], has been associated with IBD [11, 12, 67]. Consistent with the presumed role for NLRP6 in inflammasome signaling, Chen and coworkers have shown that mice lacking NLRP6 present decreased levels of serum IL-18 after DSS treatment [11]. These mice deficient in NLRP6 develop a colitis phenotype, and this is transmissible to cohoused wild-type mice, both early in postnatal life and during adulthood [67]. Upon injury, NLRP6 deficiency deregulates regeneration of the colonic mucosa and epithelial proliferation and migration. Consistently, an analysis on a whole-genome expression profiling revealed a link between NLRP6 and self-renewal of the epithelium [12]. The inability of mice lacking NLRP6 to repair damaged epithelium as efficiently as WT mice resulted in extended increase in epithelial proliferative activity [11].

Recently, the role of the inflammasome in gut-related infection and sepsis has also been addressed. For this purpose, it has been shown that mice treated with large-spectrum antibiotics before DSS intervention show symptoms of sepsis, not colitis, due to translocation of a pathogenic strain of *E. coli* [69]. This particular model is very significant due to its resemblance to the common clinical scenario in which patients undergoing antibiotic and gut-damaging cytotoxic treatments develop septicemia. In this antibiotics-DSS model, mice lacking NAIP5-NLRC4 presented highly attenuated disease progression when compared to controls. Similarly, caspase 1 and IL-1*β* deficient animals were protected from *E-coli* systemic inflammatory response showing that NAIP5-NLRC4 inflammasome signaling through IL-1*β* is important for the development of gut-related sepsis [69]. Locally, NLRC4-dependent IL-1*β* production by intestinal phagocytes represents a specific response discriminating pathogenic from commensal bacteria and contributes to host defense in the intestine [70]. Upon infection with pathogenic bacteria, intestinal phagocytes produce mature IL-1*β* through the NLRC4 inflammasome and mice deficient in NLRC4 or IL-1*β* receptor are highly susceptible to intestinal infection [70, 71]. It seems, however, that the inflammasome does not only signal through IL-1*β* or IL-18 in systemic inflammatory responses [72]. It has been shown that systemic inflammasome activation by flagellin leads to loss of vascular fluid into the intestine and peritoneal cavity and death in mice, and this outcome depends on NAIP5, NLRC4, and caspase-1 signaling, but is independent of IL-1*β* or IL-18 [72]. Instead, flagellin-related inflammasome activation results in a pathological release of signaling lipids, including prostaglandins and leukotrienes that rapidly initiate inflammation and vascular fluid loss. 

## 4. Inflammasome and Colorectal Cancer

### 4.1. Colitis-Associated Tumorigenesis

The role of the inflammasome in cancer physiopathology is complex as it can either lead to inflammasome-dependent carcinogenic inflammation or play a role in the process of eliminating malignant precursors through programmed cell death [73]. Not only the product of the inflammasome activation, caspase-1, is associated with inflammation and carcinogenesis, but also it can stimulate immune responses against tumoral cells. In colonic tissue, the role of the inflammasome in colorectal cancer tumorigenesis was mainly explored using the azoxymethane (AOM) DSS model in which administration of DSS after initiation with a low dose of AOM exerts a powerful tumor-promoting inflammatory activity in colon in mice [74]. Using this inflammation-driven tumorigenesis model draws a parallel to the carcinogenic process that takes place in IBD-related intestinal neoplasia. In these models, it has been shown that the absence of inflammasome-related interleukins, mainly IL-18, can greatly impact carcinogenesis and tumor progression. IL-18-deficient mice, for instance, have increased inflammation and tumor development in a colitis-associated colon cancer model [8]. It seems, however, that IL-18 can also influence epithelial growth by regulating the production of additional interleukins. In this regard, activation of NLRP3 or NLRP6 inflammasomes leads to IL-18-dependent downregulation of IL-22 blocking protein (IL-22bp) and higher expression of IL-22. This IL-22-IL-22bp axis was shown to critically regulate intestinal tissue repair and tumorigenesis in the colon [75]. The main studies evaluating the role of the inflammasome in colitis-associated cancer using the AOM/DSS model are summarized in Table 1.

Mice lacking NLRP3 were shown to be more susceptible to tumorigenesis in the AOM-DSS model in some studies [7, 9], but not in others [10]. In studies that demonstrated a positive association, NLRP3 deficient mice presented more inflammation and higher tumor burden compared to controls. In these NLRP3 knockouts, colonic IL-18 levels were shown to be lower than those of controls. It was postulated, therefore, that IL-18 might be associated with colon protection against tumorigenesis. In this regard, knockout mice for IL-18 treated with AOM/DSS contained significantly more tumors than controls [7, 8]. Importantly, recombinant IL-18 was successfully used as rescue, being able to reverse disease progression perhaps through induction of IFN-*γ* and its antitumor signaling involving activation of the transcription factor STAT1 [7]. Of note, IL-18 uses MyD88 as a downstream signal transduction effector and MyD88 signaling has been shown to have a protective role in the development of AOM/DSS colitis [8]. It has been proposed that the increased susceptibility of IL-18 deficient mice to colitis and cancer in the AOM/DSS model may be partially dependent on MyD88-related mechanisms, although Il-18 deficient mice present a milder phenotype compared with Myd88 knockout mice (less tumorigenesis) implying that other MyD88-related pathways might act with IL-18 to minimize carcinogenesis [8]. 

In the negative study, there were no differences in tumor formation between NLRP3 deficient mice and controls after challenge with AOM-DSS [10]. In contrast, another inflammasome, NLRC4, was found to be associated with tumorigenesis in this model. In this regard, NLRC4 knockout mice had significantly increased tumor numbers and tumor load compared to wild-type animals, though no differences in inflammation severity were noted. Since NLRC4 is associated with p53-dependent apoptosis, it may provide a link to the increased tumorigenesis observed in caspase-1 deficient mice noted by three independent groups [7, 9, 10]. Caspase-1 has been shown to be associated with the regulation of colonic epithelial cell proliferation and apoptosis and not only inflammation per se. As a result, caspase-1 deficient mice show increased colonic epithelial cell proliferation in early stages of tumor formation and reduced apoptosis in advanced tumors [10]. Hu and colleagues studied caspase-1 mRNA expression levels in normal colon tissue and colon tumors from WT mice observing a significant reduction in caspase-1 mRNA expression levels in tumors compared to normal colonic tissue, suggesting that lack of caspase-1 may play a role in tumor progression [10]. Similarly to caspase-1 deficient mice, NLRC4 knockout mice features significantly enhanced proliferation in both steady state and the early phase of inflammation-induced tumor formation [10]. 

Another inflammasome, NLRP6, was also found to play a role in AOM-DSS tumorigenesis [11, 12]. In this regard, NLRP6-deficient mice developed significantly more tumors compared to wildtype mice after chemical induction. The increase in tumors in these mice correlated with higher levels of intestinal epithelial proliferation, hyperplasia, and an increase in proinflammatory cytokines such as TNF*α*, IL-6, and IL-1*β*. Protection against tumorigenesis by NLRP6 is conferred specifically by hematopoietic cells rather than intestinal epithelial or stromal cells as irradiated wildtype mice that were transplanted with NLRP6 deficient bone marrow had similar numbers of tumors as NLRP6 deficient mice. Additionally, NLRP6 deficient recipients that received wildtype bone marrow were significantly protected against tumorigenesis to a similar extent as wildtype animals [11]. These findings suggest that deficiency in NLRP6 function in hematopoietic-derived cells is important for NLRP6-mediated protection against colitis-induced tumorigenesis.

As sporadic and familial colorectal cancer tumorigenesis in humans is often caused by Wnt-activating mutations, Normand and colleagues performed a transcriptional profiling of tumoral and nontumoral biopsies from NLRP6 deficient mice and controls treated with the DSS-AOM regimen [12]. Within the set of 1,884 genes that were differentially expressed in NLRP6 deficient mice, a significant overrepresentation of paracrine actors of the p53 Wnt and Notch signaling pathways was observed, supporting the role of NLRP6 in regulation of intestinal crypt cell proliferation. Notably, the microarray analysis clearly revealed an overexpression of Wnt-signaling pathway genes in tumor resection specimens of NLRP6 deficient mice, particularly the proto-oncogene Mycl1.

### 4.2. The Inflammasome in the ApcMin Model

In mice derived from animals treated with ethylnitrosourea, a mutation was identified that predisposed to the development of spontaneous intestinal cancer [76]. This mutation was later found to be located in the APC gene, the mouse homologue of the human APC gene responsible for human familial adenomatous polyposis [77]. The development of the APC deficient mice was one of the first spontaneous genetic animal models for bowel cancer [78]. In this model, affected mice develop multiple adenomas throughout the entire intestinal tract at an early age. It has been shown that innate immune signaling has an important role in the intestinal tumorigenesis in this model. In this regard, Rakoff-Nahoum and Medzhitov have shown that MyD88-dependent signaling controls the expression of several modifier genes of intestinal tumorigenesis in ApcMin mice [79]. ApcMin mice that are also deficient in MyD88 have decreased number of polyps which are smaller in size than those in age-matched ApcMin mice. In the inflammasome field, there were attempts to evaluate the potential impact of caspase-1 signaling in the development of tumors in ApcMin mice with disappoints results as the crossbreeding between ApcMin and caspase-1 deficient mice does not impact the phenotype [80].

## 5. Conclusion

In summary, the activation of specific NLR inflammasomes was shown to be triggered by microbial molecules, whereas defects in NLRs determine innate immune system abnormalities and changes in the intestinal microbiota. In particular, intestinal dysbiosis has been consistently linked to intestinal inflammation through defects of NLR family members. In conjunction, these data highlight the importance of the inflammasome in the innate intestinal immune response and the maintenance of intestinal homeostasis, with fundamental influence on barrier function and the efficient elimination of invading microorganisms. Therefore, the abnormal activation of the inflammasome, converging signals from the internal and external milieu, sensing diverse stressful and microbial elements, appears to position inflammasome as a critical mechanistic link in the context of chronic inflammatory disorders involving the gut.

## Figures and Tables

**Table 1:** Studies evaluating the role of the inflammasome in colitis-associated cancer using the AOM/DSS model.

Mice model	Background	Impact on cancer	Description	Publication
IL-18−/−	C57BL/6	Yes	Enhanced tumorigenesis	Zaki et al. [7] Salcedo et al. [8]
IL-18R−/−	C57BL/6	Yes	Enhanced tumorigenesis	Salcedo et al. [8]
IL-1R−/−	C57BL/6	No	No enhanced tumorigenesis	Salcedo et al. [8]
MyD88−/−	C57BL/6	Yes	Enhanced tumorigenesis	Salcedo et al. [8]
Caspase-1−/−	C57BL/6	Yes	Enhanced tumorigenesis	Zaki et al. [7] Allen et al. [9] Hu et al. [10]
ASC−/−	C57BL/6	Yes	Enhanced tumorigenesis	Zaki et al. [7]
Pycard−/−	C57BL/6	Yes	Enhanced tumorigenesis	Allen et al. [9]
NRRP-3−/−	C57BL/6	Yes	Enhanced tumorigenesis	Zaki et al. [7] Allen et al. [9]
NRRP-3−/−	C57BL/6	No	No enhanced tumorigenesis	Hu et al. [10]
NLRC4−/−	C57BL/6	Yes	Enhanced tumorigenesis	Hu et al. [10]
NLRP-6−/−	C57BL/6	Yes	Enhanced tumorigenesis	Chen et al. [11] Normand et al. [12]
